# VEGF-independent angiogenic pathways induced by PDGF-C

**DOI:** 10.18632/oncotarget.141

**Published:** 2010-08-04

**Authors:** Xuri Li, Anil Kumar, Fan Zhang, Chunsik Lee, Yang Li, Zhongshu Tang, Pachiappan Arjunan

**Affiliations:** National Eye Institute, NIH, Rockville, MD, 20852, USA

**Keywords:** PDGF-C, angiogenesis, VEGF, choroidal neovascularization

## Abstract

VEGF is believed to be a master regulator in both developmental and pathological angiogenesis. The role of PDGF-C in angiogenesis, however, is only at the beginning of being revealed. We and others have shown that PDGF-C is a critical player in pathological angiogenesis because of its pleiotropic effects on multiple cellular targets. The angiogenic pathways induced by PDGF-C are, to a large extent, VEGF-independent. These pathways may include, but not limited to, the direct effect of PDGF-C on vascular cells, the effect of PDGF-C on tissue stroma fibroblasts, and its effect on macrophages. Taken together, the pleiotropic, versatile and VEGF-independent angiogenic nature of PDGF-C has placed it among the most important target genes for antiangiogenic therapy.

## INTRODUCTION

Since its discovery ten years ago as the third member of the platelet-derived growth factor (PDGF) family [1, 2], PDGF-C has been shown to play critical roles in many biological processes. Indeed, this is consistent with its general expression profile in most of the tissues and cell types investigated [1, 3]. PDGF-C is produced as a latent protein and requires proteolytic processing for receptor binding and activation [4]. Once activated, PDGF-C binds to the PDGFR-a homodimer and the PDGFR-a/β heterodimer [1, 3, 5-7]. PDGF-C is critically required for embryonic development, since PDGF-C deficient mice die postnatally due to developmental defects when the mice are bred on a 129 background [8]. We have recently shown that PDGF-C is a critical survival factor for different types of neurons [9], although it is recently reported that PDGF-C induced blood-brain barrier permeability during ischemic stroke [10, 11]. Moreover, the role of PDGF-C in tumor growth has been shown by several groups. PDGF-C promotes tumor growth via several mechanisms. First, PDGF-C is a transforming factor [12, 13]. Second, PDGF-C is a survival and mitogenic factor for tumor cells [14]. Third, PDGF-C is a mitogenic and chemoattractant factor for cancer-associated fibroblasts [15, 16]. Fourth, PDGF-C promotes tumor angiogenesis [16, 17]. Considerable amount of research interests have been focused on the angiogenic activity of PDGF-C. Independent studies from different laboratories have shown that PDGF-C is a potent angiogenic factor in different model systems [5, 6, 16, 18, 19]. However, it remains less discussed to what extent the angiogenic activity of PDGF-C is VEGF-independent or -dependent. In this perspective, we mainly discuss the angiogenic properties of PDGF-C in relation to the VEGF-independent angiogenic pathways induced by it, and the cellular components involved in these pathways.

### PDGF-C is angiogenic in different models

PDGF-C is abundantly expressed in most of the highly angiogenic tissues, such as the placenta, ovary and embryo tissues [1, 3, 20]. Indeed, the angiogenic activity of PDGF-C occurs in many different organs and tissues. Infusion of PDGF-C protein alone increased the revascularization of ischemic mouse hearts, and induced angiogenesis in mouse ischemic hind limb [6]. In addition, the angiogenic activity of PDGF-C is comparable to that of VEGF in different model systems, such as in the aortic ring assay, chorioallantoic membrane assay and cornea pocket assay [3, 5]. The potent angiogenic activity of PDGF-C is linked to its effects on endothelial progenitor cells, bone marrow cells and mature vascular cells by promoting their recruitment, proliferation, differentiation and migration respectively [6, 7]. In the eye, PDGF-C also plays critical roles in choroidal, retinal and cornea neovascularization via its effects on multiple cellular targets, such as the vascular mural and endothelial cells, macrophages, choroidal fibroblasts, and retinal pigment epithelial (RPE) cells [5, 19].

The critical role of PDGF-C in tumor angiogenesis has been documented in different types of tumors [13, 16, 17]. It is particularly important to note that in tumors expressing a high level of PDGF-C, tumor blood vessels developed efficiently even when VEGF was inhibited [16]. This demonstrated that PDGF-C does not require VEGF activity to fulfill its angiogenic function. A combination therapy using both anti-PDGF-C and anti- VEGF antibodies was more effective in inhibiting tumor angiogenesis than using anti-VEGF treatment alone [16]. Furthermore, it is noteworthy that in the PDGF- C-overexpressing tumors, the permeability of the tumor blood vessels was decreased [17], in contrast to the VEGF-induced blood vessels, which are often leaky. The fact that PDGF-C-induced blood vessels are functionally different from the ones induced by VEGF supports that PDGF-C uses different mechanisms than VEGF to build blood vessels.

### Broad range of cellular targets of PDGF-C

One important functional characteristic of PDGF-C is that it has a considerably broad range of cellular targets (Table 1). We have shown that PDGF-C promotes the proliferation, survival and migration of vascular pericytes, endothelial cells and fibroblasts [19]. Several groups have shown that PDGF-C has direct effects on macrophages. PDGF-C regulates gene expression in macrophages [19], and promotes their migration [21] and proliferation [22]. Moreover, PDGF-C induces proliferation and migration of retinal pigment epithelial cells [23]. PDGF-C also promotes the proliferation, survival and migration of vascular endothelial cells, smooth muscle cells (SMC) and their progenitors [6, 16]. In tumors, PDGF-C plays a critical role in recruiting fibroblasts associated with tumor drug resistance [15, 16, 24]. Vascular cells are the most important cellular components in pathological angiogenesis. In addition, other cell types, such as fibroblasts, macrophages and retinal pigment epithelial cells also play critical roles in different types of pathological neovascularization [25, 26]. It is noteworthy that pericytes, smooth muscle cells, fibroblasts, macrophages, retinal pigment epithelial cells, mesangial cells [27] and hepatic stellate cells [28] are not typical cellular targets of VEGF, which mainly affects vascular endothelial cells. Thus, at cellular level, the angiogenic pathways induced by PDGF-C are unique and different from those induced by VEGF.

### VEGF-independent angiogenic pathways induced by PDGF-C

Much insight into the VEGF-independent angiogenic pathways induced by PDGF-C has derived from studies on tumor angiogenesis. For example, in tumors overexpressing PDGF-C, treatment with an anti- VEGFR-2 antibody, which blocks the VEGF pathway, had no effect on tumor angiogenesis, while it decreased blood vessel density in the control tumors without PDGF-C overexpression, demonstrating that the PDGF- C-induced angiogenesis was not mediated by the VEGF pathway [17]. Indeed, this notion was further supported by the findings that in VEGF-deficient fibrosarcomas, tumor angiogenesis still developed without VEGF, and the PDGFR-α-mediated recruitment of stromal fibroblasts was believed to be responsible for the persistent tumor angiogenesis [29], further affirming a VEGF-independent tumor angiogenesis induced by PDGF-C. Moreover, these observations were corroborated by the findings from yet another study showing that in certain types of tumors that are resistant to anti-VEGF therapy, PDGF-C was responsible for mediating the VEGF-independent tumor angiogenesis by modulating the angiogenic properties of tumor-associated fibroblasts [16]. In addition, it is recently reported that in the kidney, PDGF-C induced proliferation of glomerular endothelial cells in a VEGF- independent manner [22], indicating that the VEGF- independent effect of PDGF-C is likely a general event. Indeed, more evidence of the VEGF-independent angiogenic pathways induced by PDGF-C comes from studies on pathological ocular angiogenesis, such as choroidal and retinal neovascularization [19]. In both a laser-induced choroidal neovascularization (CNV) model and an ischemia-induced retinal angiogenesis model, the expression level of PDGF-C increased markedly. In these models, PDGF-C inhibition affected not only vascular mural and endothelial cells, but also macrophages, choroidal fibroblasts and retinal pigment epithelial cells, which are not prototypical cellular targets of VEGF. At gene regulation level, PDGF-C significantly upregulated the expression of PDGF-B and PDGF receptors in macrophages and retinal pigment epithelial cells, and PDGF-C inhibition downregulated their expression [19]. PDGF-B is known to be one of the most potent stimuli of pathological angiogenesis [30-34]. These findings indicate that PDGF-C may be functionally related to the PDGF-B-induced angiogenic pathways.

**Table 1.** Cellular targets of PDGF-C

Cell types	References
Vascular endothelial cell	[18], [6] & [16]
Vascular smooth muscle cell	[18] & [3]
Vascular pericyte	[18] & [3]
Fibroblast	[18], [3], [15] & [16]
Macrophage	[21]
Retinal pigment epithelium	[23]
Neuron	[9]
Vascular progenitor cell	[6]
Tumor cell	[12], [35] & [14]
Glomerular endothelial cell	[22]
Mesangial cell	[27]
Hepatic stellate cell	[28]

### Taken together, accumulating data have shown that PDGF-C can induce VEGF-independent angiogenesis via at least three interrelated pathways:

**Figure 1:**
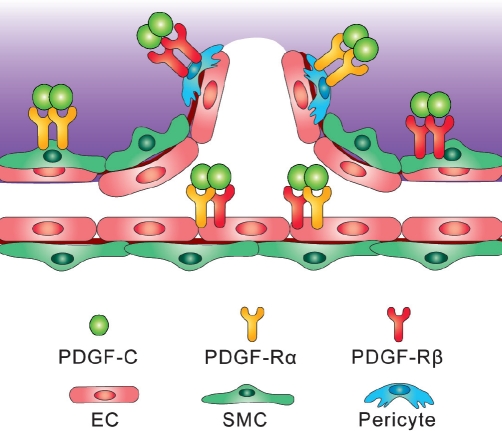
Effect of PDGF-c on three types of vascular cells. PDGF-C promotes the proliferation, survival and migration of all three types of vascular cells: endothelial cells (EC), pericytes and smooth muscle cells (SMC). Vascular pericytes and smooth muscle cells are known to be classic cellular targets of the PDGFs. In addition, certain types of vascular endothelial cells express the PDGF receptors and respond to PDGF-C directly. PDGF-C thus can induce angiogenesis by increasing the number and availability of all the three types of vascular cells.

**Figure 2:**
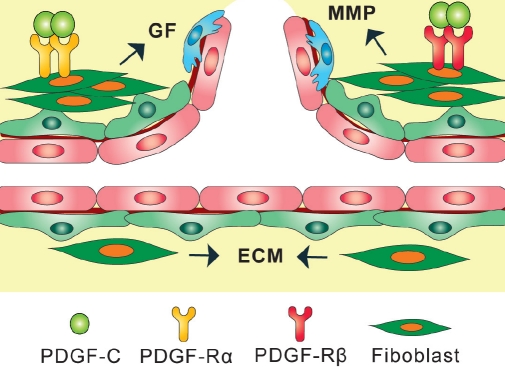
Effect of PDGF-C on fibroblasts. Fibroblasts are the principal component of mesenchymal cells and are a major source of host-derived angiogenic growth factors (GF), extracellular matrix (ECM), and ECM-degrading proteases, such as the matrix metalloproteinases (MMP). Under pathological conditions, the number of fibroblasts is increased due to the upregulated expression of chemotactant and mitogenic factors. PDGF-C is one of the most potent stimuli of fibroblast proliferation, migration and recruitment. Part of the angiogenic activity of PDGF-C is therefore exerted via its effect on fibroblasts.

**Figure 3:**
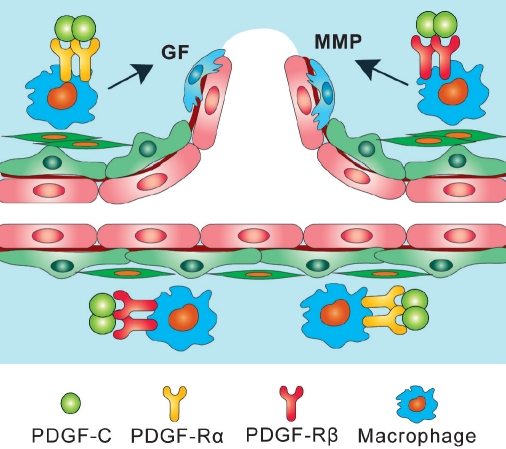
Effect of PDGF-c on macrophages. Macrophages play critical roles in VEGF-independent pathological angiogenesis by producing a broad array of angiogenic growth factors (GF), cytokines and proteolytic proteinases, such as the matrix metalloproteinases (MMP). Macrophages are required for the angiogenic switch of blood vessels in pathological conditions, and accumulation of macrophages accelerates pathological angiogenesis. Several groups have reported that PDGF-C regulates macrophage migration, proliferation and gene expression. PDGF-C therefore can promote angiogenesis via its effect on macrophages.

(1) The vascular cell pathway: PDGF-C has direct effects on all three types of vascular cells, endothelial cells, pericytes and smooth muscle cells, by promoting their proliferation, survival and migration. Vascular pericytes and smooth muscle cells are classic cellular targets of the PDGFs [35]. In addition, certain types of vascular endothelial cells express the PDGF receptors [6, 36, 37] and respond to PDGF-C stimulation directly. PDGF-C thus can induce new blood vessel formation by increasing the number and availability of all three types of vascular cells (Fig. 1).

(2) The tissue stroma pathway: Tissue stroma consists of blood vessels, extracellular matrix (ECM), mesenchymal cells, inflammatory cells, nerves, lymphatic vessels, etc. These components form a scaffold to support and promote new blood vessels to grow. Fibroblasts are the major component of mesenchymal cells and are one of the major sources of angiogenic growth factors, extracellular matrix, and ECM-degrading proteases such as the matrix metalloproteinases (MMPs). Fibroblasts can also regulate inflammation and epithelial differentiation. There are a minimal number of fibroblasts in normal stroma in most organs. However, under pathological conditions, the number of fibroblasts increases due to the upregulated expression of chemotactant and mitogenic factors. Because of their critical contributions to pathological angiogenesis, fibroblasts may represent an important cellular target in antiangiogenic therapy [38]. PDGF-C has been shown to be one of the most potent stimuli for fibroblast proliferation, migration and recruitment [1, 3, 39]. A significant part of the angiogenic activity of PDGF-C is therefore exerted via its effect on fibroblasts (Fig. 2).

(3) The inflammatory cell pathway: It is known that different types of inflammatory cells, such as macrophages, monocytes and neutrophils, play critical roles in the VEGF-independent pathological angiogenesis by producing a broad array of angiogenic growth factors and cytokines, generating conduits for new blood vessels through proteolytic mechanisms, and promoting the remodeling of arterioles into arteries [40, 41]. Particularly, macrophages are known to play a key role in promoting pathological angiogenesis in the retina and choroid in the eye [42]. Macrophages are required for the angiogenic switch of quiescent blood vessels in pathological conditions, and accumulation of macrophages accelerates this process [43]. Several groups have reported that PDGF-C is a potent regulator of macrophage migration, proliferation and gene expression [19, 21, 22, 44]. PDGF-C therefore can promote angiogenesis via its direct effect on macrophages (Fig. 3).

After being intensively studied for about two decades, VEGF has been commonly viewed as a master angiogenic factor in both developmental and pathological angiogenesis [45, 46]. The importance of PDGF-C in neovessel formation, however, is only at the beginning of being realized. The function of PDGF-C in developmental angiogenesis still needs to be explored [8]. The role of PDGF-C in pathological angiogenesis, by contrast, has been better investigated [5, 6, 16, 18, 19]. In many models, the angiogenic effect of PDGF-C was as potent as that of VEGF, such as in the cornea [5], ischemic heart and limb [6]. Moreover, in pathological ocular angiogenesis, PDGF-C inhibition suppressed neovascularization to a similar degree as VEGF inhibition [19]. Thus, at least under certain pathological conditions, PDGF-C is as potent as VEGF in inducing angiogenesis, and is therefore as critical as VEGF to be inhibited when the growth of new blood vessels is harmful.

## CONCLUSION

We and other groups have provided ample evidence showing that PDGF-C is a critical regulator of pathological angiogenesis. The cellular targets of PDGF-C are considerably broad. The angiogenic pathways induced by PDGF-C are, to a large extent, VEGF-independent. These pathways may include, but not limited to: (1) direct effect of PDGF-C on different types of vascular cells, (2) effect of PDGF-C on tissue stroma formation, particularly, on fibroblast recruitment, proliferation and migration, (3) effect of PDGF-C on inflammatory cells, especially, on macrophages. The pleiotropic, versatile and VEGF- independent angiogenic nature of PDGF-C has placed it among the most critical target genes for antiangiogenic therapy. Anti-PDGF-C treatment may prove to be therapeutically valuable in treating neovascular diseases. Combination therapy aimed at inhibiting VEGF and PDGF-C simultaneously might provide better efficacy in inhibiting undesired blood vessel growth.

## Abbreviations used:

PDGF-C: Platelet-derived growth factor-C
VEGF: vascular endothelial growth factor
PC: pericyte
SMC: smooth muscle cell
EC: endothelial cell
PDGFR: platelet-derived growth factor receptor
CNV: choroidal neovascularization
VEGFR: vascular endothelial growth factor receptor
RPE: retinal pigment epithelial cell
ECM: extracellular matrix
MMP: matrix metalloproteinase

## References

[1] Li X, Pontén A, Aase K, Karlsson L, Abramsson A, Uutela M, Bäckström G, Hellström M, Boström H, Li H, Soriano P, Betsholtz C, Heldin CH, Alitalo K, Ostman A, Eriksson U (2000). PDGF-C is a new protease-activated ligand for the PDGF alpha-receptor. Nat Cell Biol.

[2] Kazlauskas A (2000). A new member of an old family. Nat Cell Biol.

[3] Gilbertson DG, Duff ME, West JW, Kelly JD, Sheppard PO, Hofstrand PD, Gao Z, Shoemaker K, Bukowski TR, Moore M, Feldhaus AL, Humes JM, Palmer TE, Hart CE (2001). Platelet-derived growth factor C (PDGF-C), a novel growth factor that binds to PDGF alpha and beta receptor. J Biol Chem.

[4] Fredriksson L, Li H, Fiebe C, Li X, Eriksson U (2004). Tissue plasminogen activator is a potent activator of PDGF-CC. Embo J.

[5] Cao R, Brakenhielm E, Li X, Pietras K, Widenfalk J, Ostman A, Eriksson U, Cao Y (2002). Angiogenesis stimulated by PDGF-CC, a novel member in the PDGF family, involves activation of PDGFR-αα and -αβ receptors. Faseb J.

[6] Li X, Tjwa M, Moons L, Fons P, Noel A, Ny A, Zhou JM, Lennartsson J, Li H, Luttun A, Pontén A, Devy L, Bouché A, Oh H, Manderveld A, Blacher S, Communi D, Savi P, Bono F, Dewerchin M, Foidart JM, Autiero M, Herbert JM, Collen D, Heldin CH, Eriksson U, Carmeliet P (2005). Revascularization of ischemic tissues by PDGF-CC via effects on endothelial cells and their progenitors. J Clin Invest.

[7] Dimmeler S (2005). Platelet-derived growth factor CC--a clinically useful angiogenic factor at last?. N Engl J Med.

[8] Ding H, Wu X, Boström H, Kim I, Wong N, Tsoi B, O’Rourke M, Koh GY, Soriano P, Betsholtz C, Hart TC, Marazita ML, Field LL, Tam PP, Nagy A (2004). A specific requirement for PDGF-C in palate formation and PDGFR- alpha signaling. Nat Genet.

[9] Tang Z, Arjunan P, Lee C, Li Y, Kumar A, Hou X, Wang B, Wardega P, Zhang F, Dong L, Zhang Y, Zhang SZ, Ding H, Fariss RN, Becker KG, Lennartsson J, Nagai N, Cao Y, Li X (2010). Survival effect of PDGF-CC rescues neurons from apoptosis in both brain and retina by regulating GSK3beta phosphorylation. J Exp Med.

[10] Su EJ, Fredriksson L, Geyer M, Folestad E, Cale J, Andrae J, Gao Y, Pietras K, Mann K, Yepes M, Strickland DK, Betsholtz C, Eriksson U, Lawrence DA (2008). Activation of PDGF-CC by tissue plasminogen activator impairs blood- brain barrier integrity during ischemic stroke. Nat Med.

[11] Rieckmann P (2008). Imatinib buys time for brain after stroke. Nat Med.

[12] Zwerner JP, May WA (2001). PDGF-C is an EWS/FLI induced transforming growth factor in Ewing family tumors. Oncogene.

[13] Li H, Fredriksson L, Li X, Eriksson U (2003). PDGF-D is a potent transforming and angiogenic growth factor. Oncogene.

[14] Lokker NA, Sullivan CM, Hollenbach SJ, Israel MA, Giese NA (2002). Platelet-derived growth factor (PDGF) autocrine signaling regulates survival and mitogenic pathways in glioblastoma cells: evidence that the novel PDGF-C and PDGF-D ligands may play a role in the development of brain tumors. Cancer Res.

[15] Anderberg C, Li H, Fredriksson L, Andrae J, Betsholtz C, Li X, Eriksson U, Pietras K (2009). Paracrine signaling by platelet-derived growth factor-CC promotes tumor growth by recruitment of cancer-associated fibroblasts. Cancer Res.

[16] Crawford Y, Kasman I, Yu L, Zhong C, Wu X, Modrusan Z, Kaminker J, Ferrara N (2009). PDGF-C mediates the angiogenic and tumorigenic properties of fibroblasts associated with tumors refractory to anti-VEGF treatment. Cancer Cell.

[17] di Tomaso E, London N, Fuja D, Logie J, Tyrrel JA, Kamoun W, Munn LL, Jain RK (2009). PDGF-C induces maturation of blood vessels in a model of glioblastoma and attenuates the response to anti-VEGF treatment. PLoS ONE.

[18] Campbell JS, Johnson MM, Bauer RL, Hudkins KL, Gilbertson DG, Riehle KJ, Yeh MM, Alpers CE, Fausto N (2007). Targeting stromal cells for the treatment of platelet-derived growth factor C-induced hepatocellular carcinogenesis. Differentiation.

[19] Hou X, Kumar A, Lee C, Wang B, Arjunan P, Dong L, Maminishkis A, Tang Z, Li Y, Zhang F, Zhang SZ, Wardega P, Chakrabarty S, Liu B, Wu Z, Colosi P, Fariss RN, Lennartsson J, Nussenblatt R, Gutkind JS, Cao Y, Li X (2010). PDGF-CC blockade inhibits pathological angiogenesis by acting on multiple cellular and molecular targets. Proc Natl Acad Sci USA.

[20] Li X, Eriksson U (2003). Novel PDGF family members: PDGF-C and PDGF-D. Cytokine Growth Factor Rev.

[21] Wagsate D, Zhu C, Bjorck HM, Eriksson P (2009). Effects of PDGF-C and PDGF-D on monocyte migration and MMP-2 and MMP-9 expression. Atherosclerosis.

[22] Boor P, van Roeyen CR, Kunter U, Villa L, Bücher E, Hohenstein B, Hugo CP, Eriksson U, Satchell SC, Mathieson PW, Eitner F, Floege J, Ostendorf T (2010). PDGF-C mediates glomerular capillary repair. Am J Pathol.

[23] Li R, Maminishkis A, Wang FE, Miller SS (2007). PDGF-C and-D Induced Proliferation/Migration of Human RPE Is Abolished by Inflammatory Cytokines. Invest Ophthalmol Vis Sci.

[24] Francia G, Emmenegger U, Kerbel RS (2009). Tumor-associated fibroblasts as “Trojan Horse” mediators of resistance to anti-VEGF therapy. Cancer Cell.

[25] Kvanta A (1995). Expression and regulation of vascular endothelial growth factor in choroidal fibroblasts. Curr Eye Res.

[26] Motiejunaite R, and Kazlauskas A (2008). Pericytes and ocular diseases. Exp Eye Res.

[27] Eitner F, Ostendorf T, Van Roeyen C, Kitahara M, Li X, Aase K, Grone HJ, Eriksson U, Floege J (2002). Expression of a Novel PDGF Isoform, PDGF-C, in Normal and Diseased Rat Kidney. J Am Soc Nephrol.

[28] Breitkopf K, Roeyen C, Sawitza I, Wickert L, Floege J, Gressner AM (2005). Expression patterns of PDGF-A, -B, -C and -D and the PDGF-receptors alpha and beta in activated rat hepatic stellate cells (HSC). Cytokine.

[29] Dong J, Grunstein J, Tejada M, Peale F, Frantz G, Liang WC, Bai W, Yu L, Kowalski J, Liang X, Fuh G, Gerber HP, Ferrara N (2004). VEGF-null cells require PDGFR alpha signaling-mediated stromal fibroblast recruitment for tumorigenesis. Embo J.

[30] Jo N, Mailhos C, Ju M, Cheung E, Bradley J, Nishijima K, Robinson GS, Adamis AP, Shima DT (2006). Inhibition of platelet-derived growth factor B signaling enhances the efficacy of anti-vascular endothelial growth factor therapy in multiple models of ocular neovascularization. Am J Pathol.

[31] Akiyama H, Kachi S, Silva RL, Umeda N, Hackett SF, McCauley D, McCauley T, Zoltoski A, Epstein DM, Campochiaro PA (2006). Intraocular injection of an aptamer that binds PDGF-B: A potential treatment for proliferative retinopathies. J Cell Physiol.

[32] Kodama T, Oku H, Kawamura H, Sakagami K, Puro DG (2001). Platelet-derived growth factor-BB: a survival factor for the retinal microvasculature during periods of metabolic compromise. Curr Eye Res.

[33] Nissen LJ, Cao R, Hedlund EM, Wang Z, Zhao X, Wetterskog D, Funa K, Brakenhielm E, Cao Y (2007). Angiogenic factors FGF2 and PDGF-BB synergistically promote murine tumor neovascularization and metastasis. J Clin Invest.

[34] Cao R, Brakenhielm E, Pawliuk R, Wariaro D, Post MJ, Wahlberg E, Leboulch P, Cao Y (2003). Angiogenic synergism, vascular stability and improvement of hind-limb ischemia by a combination of PDGF-BB and FGF-2. Nat Med.

[35] Andrae J, Gallini R, Betsholtz C (2008). Role of platelet-derived growth factors in physiology and medicine. Genes Dev.

[36] Edelberg JM Aird WC, Wu W, Rayburn H, Mamuya WS, Mercola M, Rosenberg RD (1998). PDGF mediates cardiac microvascular communication. J Clin Invest.

[37] Marx M, Perlmutter RA, Madri JA (1994). Modulation of platelet- derived growth factor receptor expression in microvascular endothelial cells during in vitro angiogenesis. J Clin Invest.

[38] Kalluri R, Zeisberg M (2006). Fibroblasts in cancer. Nat Rev Cancer.

[39] Jinnin M, Ihn H, Mimura Y, Asano Y, Yamane K, Tamaki K (2005). Regulation of fibrogenic/fibrolytic genes by platelet- derived growth factor, C, a novel growth factor, in human dermal fibroblasts. J Cell Physiol.

[40] Ferrara N (2010). Role of myeloid cells in vascular endothelial growth factor-independent tumor angiogenesis. Curr Opin Hematol.

[41] David Dong ZM, Aplin AC, Nicosia RF (2009). Regulation of angiogenesis by macrophages, dendritic cells, and circulating myelomonocytic cells. Curr Pharm Des.

[42] Apte RS (2010). Regulation of angiogenesis by macrophages. Adv Exp Med Biol.

[43] Qian BZ, Pollard JW (2010). Macrophage diversity enhances tumor progression and metastasis. Cell.

[44] Eitner F, Bücher E, van Roeyen C, Kunter U, Rong S, Seikrit C, Villa L, Boor P, Fredriksson L, Bäckström G, Eriksson U, Ostman A, Floege J, Ostendorf T (2008). PDGF-C is a proinflammatory cytokine that mediates renal interstitial fibrosis. J Am Soc Nephrol.

[45] Leung DW, Cachianes G, Kuang WJ, Goeddel DV, Ferrara N (1989). Vascular endothelial growth factor is a secreted angiogenic mitogen. Science.

[46] Chung AS, Lee J, Ferrara N (2010). Targeting the tumour vasculature: insights from physiological angiogenesis. Nat Rev Cancer.

